# The relationship between problem gambling, excessive gaming, psychological distress and spending on loot boxes in Aotearoa New Zealand, Australia, and the United States—A cross-national survey

**DOI:** 10.1371/journal.pone.0230378

**Published:** 2020-03-23

**Authors:** Aaron Drummond, James D. Sauer, Christopher J. Ferguson, Lauren C. Hall

**Affiliations:** 1 School of Psychology, Massey University, Palmerston North, Manawatu, Aotearoa New Zealand; 2 International Media Psychology Laboratory, University of Tasmania, Hobart, Australia; 3 Psychology, School of Medicine, University of Tasmania, Hobart, Australia; 4 Department of Psychology, Stetson University, DeLand, Florida, United States of America; University of Auckland, NEW ZEALAND

## Abstract

Loot boxes are digital containers of randomised rewards available in many video games. Due to similarities between some loot boxes and traditional forms of gambling, concerns regarding the relationship between spending on loot boxes in video games and symptoms of problematic gambling have been expressed by policy makers and the general public. We present the first investigation of these concerns in large cross-sectional cross-national samples from three countries (Aotearoa New Zealand, Australia, and the United States). A sample of 1,049 participants were recruited through Qualtrics’ Survey Targeting service from a broad cross-section of the population in Australia (n = 339), Aotearoa New Zealand (n = 323), and the United States (n = 387). Participants answered a survey assessing problem gambling, problem gaming symptomology, and how much they spent on loot boxes per month. On average, individuals with problem gambling issues spent approximately $13 USD per month more on loot boxes than those with no such symptoms. Loot box spending was also associated with both positive and negative moods, albeit with small effect sizes. Analyses showed both interactions and correlations between problematic gambling and problematic gaming symptoms, indicating both some commonality in the mechanisms underlying, and independent contributions made by, these proposed diagnostic criteria. These results provide context for dialogues regarding how best to reduce the impacts of loot box spending among those with problematic gambling symptoms.

## Introduction

Loot boxes are randomized containers of digital rewards available in some video games, and often purchasable for real-world money. Upon purchase, loot boxes provide players with a randomized reward which might alter the appearance or gameplay of the video game they are playing. There has been growing concern that at least some loot boxes are psychologically akin to traditional forms of gambling [1]. These concerns have escalated to result in bans on loot boxes (in Belgium and the Netherlands), a requirement for companies to disclose the odds of winning particular items (in China and Japan), recommended restrictions (in Australia and the UK), and legislation to ban or regulate loot boxes being under consideration [in the US and Aotearoa New Zealand; 2, 3]. Further, in line with recommendations to help consumers make informed decisions when purchasing loot boxes [4], many games platforms have recently agreed to disclose the odds of winning high and low value items prior to the player purchasing the loot box in all countries (not just China and Japan where this is required by law).

The similarities between loot boxes and traditional forms of gambling are, to some extent, self-evident. In addition to offering rewards on a variable ratio reinforcement schedule, many loot box systems meet key psychological criteria distinguishing traditional gambling activities from other forms of risk taking [1]. They can often be bought for real money, offer rewards based on chance, and, where rewards provide players with significant in-game power-ups (in some, but not all, games; e.g., powerful weapons) they may result in players who received these rewards being more likely to defeat players who received inferior rewards (or who opted out) from purchasing loot boxes in future games. In some cases, where the parallels between loot boxes and conventional gambling are most obvious, players can cash out their virtual rewards for real world money (either by trading with other players, such as in Player Unknown’s Battlegrounds, or via third party websites; see *1* for further discussion).

These mechanistic similarities between loot boxes and conventional forms of gambling may make loot boxes especially appealing to people who have difficulty regulating their gambling behaviors (i.e., problem gamblers). For instance, players with gambling problems who are wanting to obtain rare or desirable items from loot boxes may compulsively purchase and open loot boxes in an attempt to obtain such items. The variable ratio reinforcement schedules used in loot boxes are common to gambling and, as they do in conventional gambling activities, might plausibly cause players to feel that the next opening will yield a valuable prize, resulting in increased spending [1]. This may explain why, compared to non-problem gamblers, problem gamblers spend significantly more on loot box systems [5–11]. We note, that although mean spending amongst problem gamblers is typically reported as being low, between $40–50 USD per month, this figure is drawn from research primarily involving samples of convenience and requires representative sampling to validate.

Despite the rapid increase in research investigating the relationship between loot boxes and gambling, much remains unknown about the relationship between player engagement with loot boxes and problem gambling symptomology. Most of the work in this area has been undertaken with samples self-selecting into online surveys [5], or by specifically targeting populations likely to be heavy gamers [7, 8, 10, 12].

To our knowledge, only Zendle [13] has surveyed a nationally representative sample (in this case, of UK citizens). While Zendle’s study represents an important methodological improvement, it leaves several key issues unaddressed. First, the study only examines UK citizens, leaving questions about the generalizability of these findings to other nations, or the consistency of the relationships across cultures. Studies have shown significant cultural and cross-national differences in gambling behaviours [14, 15], and it is therefore important to empirically determine the consistency of the relationship between problem gambling symptomology and loot box purchasing across countries. Moreover, as much legislation is being enacted at a national level, it is essential that policymakers have relevant national information available about the relationship between problem gambling and loot box purchasing within their jurisdictions. Here, we sought to examine three representative national samples of Australians, Aotearoa New Zealanders, and US citizens to determine the relationship between problem gambling symptoms and loot box spending, as well as the consistency of this relationship across these three countries. These three countries have similar policy making processes, making this study particularly informative to policymakers in these three countries. To foreshadow, we were unfortunately unable to obtain a representative sample. While this limits our ability to make population estimates, our final sample still allows us to examine the relationships between these psychological variables, and the consistency of these relationships across countries.

Little is known about the factors underpinning spending on loot boxes. For instance, there is debate about whether excessive spending on loot boxes is better contextualized within the framework of problem gambling [11] or excessive gaming [16]. Although there have been numerous studies highlighting relationships between problem gambling and loot box spending [5–11, 13], some argue that excessive game use could also plausibly contribute to the relationship in a meaningful way [6, 16]. Under this interpretation, having increased time investment in the activity of video gaming is thought to potentially increase the desire to obtain items from loot boxes [16], and because some loot boxes can be earned through gameplay, it is also thought that desire to obtain more loot boxes might increase playtimes exacerbating any underlying excessive gameplay [5, 16].

Here we incorporated scales of both problem gambling and gaming to examine the relationship each has with loot box spending. We also examined whether excessive gaming might act as a moderator of the relationship between problem gambling and loot box spending, such that people with high problem gambling symptomology who also have high symptoms of excessive gaming might have increased loot box spending compared to those people with higher problem gambling symptoms but fewer symptoms of excessive gaming.

## The current study

We extended the aforementioned studies in a number of ways. We report a sample from three countries, two of which have not previously been sampled on this issue–Aotearoa New Zealand (ANZ), Australia, and the United States. Given debate about whether problem loot box expenditure is best interpreted in the context of gambling symptomology [1, 11] or excessive gameplay [16], we examined whether problem gambling and problem gaming symptomology made independent contributions to predicting loot box spending. This adds to our understanding of whether problematic loot box spending is best considered within a problematic gambling or problematic gaming framework, or whether both frameworks are required. Further, Brooks and Clark [5] propose that specific high risk engagement with loot boxes (such as feeling compelled to open more loot boxes after opening a first) might be associated with increased spending on the mechanism. We thus examined the relationship between the Risky Loot Box Index (RLI, 5), loot box spending and Internet Gaming Disorder (IGD) symptomology.

Finally, our study design, hypotheses, and analyses were pre-registered (see Methods section for full details). Though many studies examining the relationship between problem gambling symptomology and loot box spending have been pre-registered, to date, no studies assessing both psychological distress and excessive gaming have been pre-registered. Here, we constrained the increased risk of Type-1 error associated with unidentified researcher degrees of freedom [17] through extensive pre-registration.

Based on the literature, we predicted results in two broad outcome categories. First, we examined the relationship between a variety of factors (e.g., problem gambling symptomology, excessive gameplay) and spending on loot boxes. Second, we examined the relationship between each of the impulse control disorder scales. That is, problematic gambling, excessive gaming, and risky loot box engagement all index impulse control problems and should therefore be empirically related. Thus, based upon the literature, we predicted the following(Note that we have re-ordered the hypotheses from the pre-registration to be clustered into hypotheses about spending behavior and impulse control disorders):

### Spending behavior

We predicted that the amount of money participants reported spending on purchasing loot boxes in the past month would correlate

1. *positively* with problem gambling symptoms as measured by the Problem Gambling Severity Index (PGSI).
2. *positively* with Risky Loot Box Spending Index [5].
3. *positively* with negative affect as measured by the Positive and Negative Affect Scale Short Form (PANAS-SF).
4. *negatively* with positive affect as measured by the PANAS-SF.
5. *positively* with psychological distress as measured by the Kessler Psychological Distress Scale (K-10).
  We also predicted that
6. Internet Gaming Disorder Symptomology would moderate the relationship between problem gambling symptomology and loot box spending such that participants who have higher problem gambling symptomology (PGSI scores) and high scores on the IGD scale, will spend significantly more than participants without high scores on both of these measures.
7. Participants who are categorized as *problem gamblers* by the PGSI would report *spending more money* in the past month on loot boxes than participants who are categorized by the PGSI to be (a) *moderate risk gamblers*, (b) *low risk gamblers* and (c) *non*-*problem gamblers*.
8. Participants who are categorized *as moderate risk gamblers* by the PGSI will report *spending more money* in the past month on loot boxes than participants who are categorized by the PGSI to be (a) *low risk gamblers* or (b) *non*-*problem gamblers*.
9. Participants who are categorized as *low risk gamblers* by the PGSI will report *spending more money* in the past month on loot boxes than participants who are categorized by the PGSI to be *non-problem gamblers*.
10. There would be no significant relationship between problem gambling symptoms and the amount participants report spending on non-randomized rewards in video games.
  **Impulse control disorders**
  We predicted positive correlations between
11. the Risky Loot Box Spending Index and scores on the Internet Gaming Disorder (IGD) questionnaire adapted from Przybylski, Weinstein & Murayama [18].
12. gambling symptoms as measured by the PGSI and scores on the Internet Gaming Disorder (IGD) questionnaire adapted from Przybylski, Weinstein & Murayama [18].

## Method

### Ethics

Ethics approval was obtained for human data collection for this study from Massey University’s Human Ethics Southern B Committee, Approval number SOB 19/11.

### Pre-registration

Our pre-registration (including planned exclusions, analysis plans and decision rules) can be accessed on the Open Science Framework Page at DOI: 10.17605/OSF.IO/B87PM.

### *A-priori* power analysis

We conducted a power analysis in G*Power. A sample size of 1200 allows us to reliably detect correlations of .1 (the smallest correlation of interest) in the total sample, and .2 in the individual country samples, with 0.95 power. As past research suggests the relationship between loot box spending and problem gambling symptomology is around .24, this allowed us to reliably detect the effects that we expect within both the overall sample, and the individual country samples. However, with our exclusions (see below) we achieved only .86% power to detect an effect at .1. Given this was above the conventional power of .8, and most of the expected effects were larger than .2, we were satisfied with our statistical power to reliably detect a small effect.

### Design

We undertook a cross-sectional between-subjects correlational design. Primary measures were problem gambling symptoms as measured by the PGSI (a continuous scale which can also be collapsed into four categories as described below); internet gaming disorder symptomology (continuous) as measured by our adapted version of Przybylski et al.’s [18] questionnaire; loot box spending in the past month (continuous) measured by self-report; positive and negative affect in the past week (continuous) as measured by the PANAS-SF; and psychological distress (continuous) as measured by the Kessler-10.

### Participants

These were three national samples recruited through Qualtrics’ Survey Targeting Tool. We requested Qualtrics’ Survey Targeting team to recruit a sample representative to the age and income demographics of the country as reported in national Census data. This resulted in 1,288 respondents, 433 from Australia, 419 from Aotearoa New Zealand, and 436 from the US. Average age was 40 years (SD = 15.4), matching the expected age demographics of the sample. Unfortunately, the data appeared not to be representative. The sample overrepresented female respondents (n = 816, 63.4%) compared to male respondents (n = 457, 35.5%). Seven participants identified as gender non-binary, 6 preferred not to disclose their gender and 2 listed their gender as “other”. The data also appeared to over-represent the proportion of problem gamblers compared to what would be expected in the general population, with 17% of the sample meeting the PGSI criteria for problem gambling. We therefore do not believe the sample was truly representative. While we are unable to make population prevalence inferences from these data, we may still examine the relationship between variables.

### Exclusions

We pre-registered a series of exclusion rules. Only three exclusion criteria were relevant to our data. These were: any participants who endorsed the statement “I once owned a three headed dog”; participants who indicated that they never played video games; and participants who were greater than 3.29 standard deviations from the mean on Loot Box Spending (equating to $104.20 USD). We excluded 22 participants who reported that they once owned a three headed dog, deeming these participants as mischievous responders as per our pre-registration documentation.

Effect sizes can be inflated by a very small number of extreme scores (outliers). To minimize these effects, we also pre-registered that we would follow the exclusion recommendation for outliers [19] of excluding anyone who had spent more than 3.29 SDs above the mean expenditure on loot boxes during the past month. This resulted in us excluding 15 participants (1.1% of the sample) who reported spending more than $104.20 USD on loot boxes in the past month. Where the exclusion of outliers alters the patterns of results, we report the analyses with and without these exclusion rules applied for completeness.

For all analyses we also excluded participants who indicated they never played video games, further reducing the sample. Including participants who never played video games in the analyses did not qualitatively alter the results. This reduced our sample by a further 202 participants.

No other exclusions were applied (i.e., no participants were excluded due to the other exclusion criteria outlined in our pre-registration document). These exclusions resulted in a final sample of 1,049 participants (339 Australians, 323 Aotearoa New Zealanders and 387 Americans).

### Variables

#### Problem gambling symptoms

Problem Gambling Symptoms were measured using the Problem Gambling Severity Index (PGSI). The PGSI asks participants to rate how often (0, never– 3, almost always) during the past 12 months, they experienced problems caused by their gambling behavior (e.g., borrowed money or sold something to get money to gamble). The instrument consists of 9 items, and has a range from 0–27 with higher scores indicating greater symptoms of problem gambling. The PGSI has been shown to have good validity in a non-clinical population [20]. The scale can also be used to categorize participants into discrete groups of varying risk for gambling problems. Participants who score 0 on the scale are considered non-problem gamblers; low risk gamblers score 1–2 on the PGSI; moderate risk gamblers = 3–7 on the PGSI and problem gambler score 8 or higher on the PGSI. Internal reliability of this scale was high (α = .936).

#### Excessive gaming

To assess excessive gaming, we adapted the Internet Gaming Disorder Checklist [18]. This checklist was based on the proposed diagnostic criteria for Internet Gaming Disorder and asked participants to indicate how true (1, not at all true– 4, very true) various statements were about how gaming had interfered with aspects of their lives (e.g., I have lost interest in other hobbies or entertainment in order to play games) or emotions (e.g., I feel irritable, anxious or sad when I am unable to game). The nine items resulted in a scale which ranged from 9–36 with higher scores indicating greater symptoms of excessive gaming. Internal reliability of this scale was high (α = .894).

#### Positive and Negative Affect Scale Short Form (PANAS-SF)

The PANAS-SF was administered to examine the positive and negative mood of participants [21]. The PANAS-SF contains 20 items, 10 measuring positive mood (Interested, Exited, Strong, Enthusiastic, Proud, Alert, Inspired, Determined, Attentive and Active) and 10 measuring negative mood (Distressed, Upset, Guilty, Scared, Hostile, Irritable, Ashamed, Nervous, Jittery and Afraid). Participants are asked the extent to which they have experienced each emotion in the past week on a scale from very slightly or not at all [1] to extremely [5]. This results in two sub-scales, one for positive emotions and one for negative emotions, each ranging from 10–50, with higher scores indicating greater positive or negative mood respectively. Internal reliability of these sub-scales was high (Positive mood, α = .902; Negative mood α = .903).

#### Psychological distress

Psychological distress was measured using the Kessler (K-10) Psychological Distress Scale [22]. This scale consists of 10 items assessing how often (0, none of the time– 5, all of the time) over the past 30 days participants experienced various non-specific aspects of psychological distress (e.g., “During the last 30 days, about how often did you feel nervous?”). The scale ranges from 10–50 with higher scores indicating greater psychological distress. Internal reliability of this scale was high (α = .934).

#### Risky loot box index

The Risky Loot Box index (RLI), is a five item scale designed to examine risky engagement with loot box mechanics [5]. The scale asks participants to rate their agreement (1, strongly disagree– 7, strongly agree) with a number of items assessing their risky cognitions associated with opening loot boxes (e.g., Once I open a loot box, I often feel compelled to open another). The scale consists of 5 items and ranges from 5–35, with higher values indicating more risky loot box engagement. Internal reliability of this scale was high (α = .915).

#### Spending on loot boxes in past month

Our critical dependent variable was spending on loot boxes. We asked participants to report approximately how much money (in their country’s currency) they had spent on loot boxes in the past month. We converted all values into US dollars on the 2^nd^ of October 2019, using the listed currency conversion rates of the day using Google’s currency conversion. For Australian purchases, this resulted in the spending data being 0.67 times the reported amount, and for ANZ purchases, this resulted in the spending data being 0.62 times the reported amount.

#### Spending on non-randomized virtual rewards in the past month

We also asked participants to report approximately how much money (in their country’s currency) they had spent on non-randomized rewards in video games in the past month. As with loot box spending, we converted all values into US dollars on the 2^nd^ of October 2019, using the listed currency conversion rates of the day. For Australian purchases, this resulted in the spending data being 0.67 times the reported amount, and for ANZ purchases, this resulted in the spending data being 0.62 times the reported amount.

## Results

Our data are publicly available for analysis at https://osf.io/b87pm/?view_only=8d23ec1a41de47ceb75d9673dee1cb9a.

### Confirmatory analyses

#### Spending behavior

Participant’s reports on the amount they had spent on loot boxes in the past month had high skewness (4.23) and Kurtosis (19.53) scores even after outliers were removed. As pre-registered, we used a Spearman rank order correlation to assess the correlation between this variable and the independent predictors. Table 1 shows the relationships between loot box spending, and the independent predictors of problem gambling symptomology, the Risky Loot Box Index, IGD Symptomology, Positive Mood, Negative Mood and psychological distress. All but two of the predicted relationships were borne out in the data, and results were qualitatively similar when outliers were included in the analyses. Further, analyses controlling for age and gender did not meaningfully alter the effect sizes: The critical relationships between loot box spending, PGSI scores and risky loot box engagement remained moderate in size.

**Table 1:** Relationships (Spearman’s rho) between loot box spending, Problem Gambling Symptomology (PGSI), the Risky Loot Box Index (RLI), IGD symptomology, positive mood, and negative mood and psychological distress (K-10). Controlled analyses are partial correlations controlling for age and gender.

	PGSI	RLI	Positive Mood	Negative Mood	K-10
**Loot Box Spend**	.344**	.391**	.148**	.182**	.169**
**Loot Box Spend (Controlled)**	.328**	.392**	.163*	.140*	.138*

*p < .01

**p < .001

The two predictions not supported were (a) greater loot box spending was not negatively associated with positive affect (in fact a positive relationship emerged), and (b) spending on non-randomized virtual rewards was, contrary to the predicted null relationship, positively associated with problem gambling symptoms. As with loot box spending, spending on non-randomized rewards also displayed high non-normality (skewness = 4.67, kurtosis = 25.52). We undertook a Spearman’s rank-order correlation on the relationship between spending on non-randomized rewards and problem gambling symptomology. There was a significant positive relationship between spending on non-randomized rewards and problem gambling symptoms, *r*_*s*_ = .361, *p* < .001. Including outliers on the non-randomized spending variable made little difference to these results. The implications of this finding will be discussed in the discussion section.

**Fig 1:**
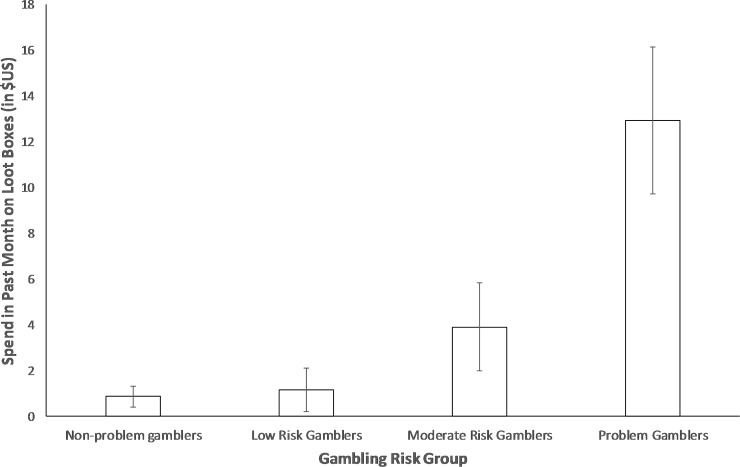
Differences in loot box spending between participants classified by the original classification scheme of the PGSI to be non-problem gamblers, low risk gamblers, moderate risk gamblers, and problem gamblers. Error bars represent 95% Confidence Intervals.

A one-way ANOVA revealed a significant difference in loot box spending based on gambling risk as classified by the PGSI, *F* (3, 1045) = 48.497, *p* < .001. Fig 1 displays loot box spending for participants classified by PGSI to be non-problem gamblers, low risk gamblers, moderate risk gamblers and problem gamblers. As Fig 1 shows, and consistent with our predictions, problem gamblers spent significantly more than moderate risk gamblers, *t* (378) = 4.57, *p* < .001, low risk gamblers, *t* (358) = 6.19, *p* < .001, and non-problem gamblers, *t* (709) = 11.00, *p* < .001. Similarly, moderate risk gamblers spent significantly more than low risk, *t* (336) = 2.40, *p* = .017, or non-problem gamblers, *t* (687) = 4.31, *p* < .001. Contrary to predictions, however, low risk gamblers and non-problem gamblers did not significantly differ in their loot box spending, *t* (667) = 0.59, *p* = .553. Results were similar when outliers were included, except that moderate risk gamblers (*M* = 7.74, *SD* = 39.04) did not significantly differ from low risk gamblers (*M* = 3.67, *SD* = 32.10), *t* (339) = 1.05, *p* = .297. All other effects were unaffected by the inclusion of outliers. It is worth noting that spending on loot boxes in the past month was, on average, reasonably low in real terms, even in the problem gambling group (*M* = $12.92, *SD* = $23.29), though the large Standard Deviation belies the long right tail in spending. It is unclear whether this low spending represents lower spending than observed in other countries (e.g., the UK [13]), or a result of our sample not being representative.

**Fig 2:**
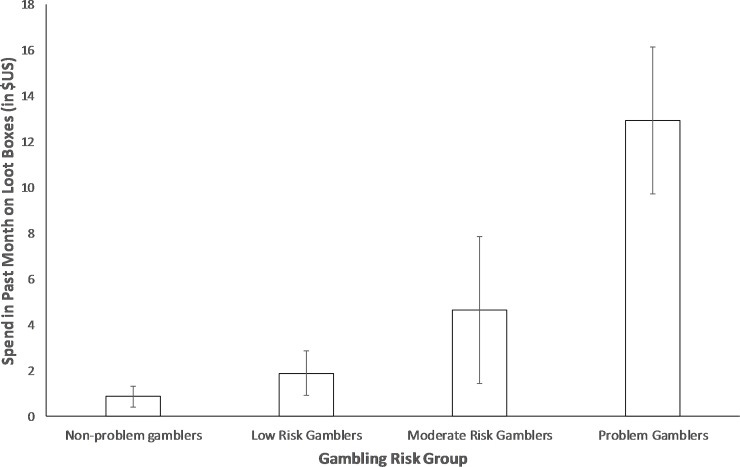
Differences in loot box spending between participants classified by the revised classification scheme of the PGSI to be non-problem gamblers, low risk gamblers, moderate risk gamblers, and problem gamblers. Error bars represent 95% Confidence Intervals.

Revised coding of the PGSI scale into categories alters the low and moderate risk gambler cut-offs to improve the discriminant validity of these categories [23]. Results were similar when this revised PGSI categorization was employed [23]. The differences between the groups using the revised coding scheme can be seen in Fig 2. Under this coding, non-problem gamblers (*M* = $0.88, *SD* = $5.24) spent significantly less than low risk gamblers (*M* = $1.88, *SD* = $7.82), t(756) = 2.10, *p* = .036, who spent less than moderate risk gamblers (*M* = $4.64, *SD* = $15.60), t(336) = 2.14, *p* = .033, who in turn spent less than problem gamblers (*M* = $12.91, *SD* = $23.29), *t*(289) = 3.07, *p =* .002. Use of this revised coding created a clearer trend for each risk-category to increase in spending, more closely approximating our theoretical predictions and the linear relationships observed in the correlational analyses and may therefore be the most appropriate categorization system for problem gambling symptomology in loot box research moving forward.

#### Moderation analysis

We hypothesized that symptoms of excessive gaming would moderate the relationship between problem gambling symptoms and loot box spending, such that those with high levels of both excessive gaming and problem gambling symptoms would spend more than those with lower symptoms in either of these issues (i.e., that there would be an additive relationship between these predictors). We deviated from our pre-registered analysis plan due to collinearity issues. Due to high collinearity (VIFs > 5.0) we centered our variables prior to computing an interaction term. The centered variables showed little collinearity (VIFs < 2.5). A stepwise regression analysis, entering IGD and PGSI symptomology at step 1 and IGD*PGSI at step 2 supported this hypothesis. There was a significant interaction between IGD and PGSI symptoms on loot box spending (ϐ = .230, *p <* .001) which was larger than for either the effect of IGD (ϐ = .187, *p* < .001) symptoms, but smaller than the effects of PGSI scores (ϐ = .267, *p* < .001) alone on spending behavior. Using the uncentered variables artificially inflated the interaction effect to ϐ = .551. People with symptoms of excessive gaming *and* problem gambling spend more on loot boxes than peers with low symptoms on one or both of these individual measures.

#### Impulse control disorders

As predicted, the measures of impulse control disorders all correlated closely. Table 2 shows the close agreement between the PGSI, Risky Loot Box Index, and Internet Gaming Disorder (IGD) scales. This suggests significant overlap in the underlying constructs being measured by each of these scales.

**Table 2:** Relationships (Pearson’s r) between impulse control disorders: Problem Gambling Symptomology (PGSI), the Risky Loot Box Index (RLI), and IGD symptomology.

	PGSI	RLI
**PGSI**	-	
**RLI**	.411**	-
**IGD**	.596**	.600**

**p < .001.

#### Exploratory analyses

We undertook an exploratory analysis of the relationships between loot box spending and problem gambling symptomology, risky loot box engagement, positive and negative affect, and psychological distress split by country. Table 3 shows these relationships. The table shows similar but slightly divergent results by country. As these analyses were not pre-registered they should be interpreted cautiously, however, they may be indicative of cultural differences in the way that people engage with loot box mechanisms. Compared to Australia and the US, Aotearoa New Zealand had a weaker association between loot box spending and problem gambling scores, but a stronger association between loot box spending and negative mood, and a stronger association between loot box spending and psychological distress. In Aotearoa New Zealand the relationship between loot box spending and psychological distress approached a Spearman’s Rho of .2, indicating a potentially clinically meaningful relationship ripe for further investigation [24]. In contrast, Australia appears to experience a larger positive association between positive mood and loot box spending, and no relationship between loot box spending and psychological distress. In both cases the US showed relationships with strengths in-between the strengths observed in the Australian and Aotearoa New Zealand samples. These results may indicate that differential responses to loot boxes are required across different countries.

**Table 3:** Relationships (Spearman’s rho) between impulse control disorders: Problem Gambling Symptomology (PGSI), the Risky Loot Box Index (RLI), positive and negative mood (PANS-SF) and IGD symptomology, split by country.

	PGSI	RLI	Positive Mood	Negative Mood	K-10
**Loot Box Spend (Australia)**	.389**	.399**	.211**	.208*	.139*
**Loot Box Spend (Aotearoa New Zealand)**	.214**	.314**	.063	.171**	.178**
**Loot Box Spend (United States)**	.365**	.421**	.148**	.134*	.151*

*p < .01

**p < .001

## Discussion

We investigated the relationships between loot box spending, problem gambling symptoms, psychological functioning, mood, and excessive gaming in samples from Australia, Aotearoa New Zealand, and the United States. In general, the results supported our predictions. Participants with higher gambling symptoms and more risky loot box related engagement spent more on loot boxes than those without. Moreover, participants with greater loot box spending experienced greater negative mood, and more psychological distress. Only two of our specific predictions were not supported. First, increased loot box spending was not associated with decreased positive mood, but with increased positive mood (albeit a small effect). Second, and surprisingly, the association between spending and problem gambling symptoms was not unique to randomized loot box purchases. People with higher problem gambling symptomology also purchased more non-randomized items. Each of these findings warrants discussion in more detail.

There are multiple reasons why a positive relationship between loot box spending and mood may exist. Recent research shows that spending on items congruent with individual’s personalities is associated with increased happiness [25]. Thus, gamers who have enough disposable income to spend money on luxuries like loot boxes might also have more disposable income to spend on other personality congruent purchases and thus be a general predictor of positive mood. By this account, it would be disposable income—not loot box spending per se–that was the antecedent of positive mood. Another possibility is that opening loot boxes is fun. The positive moods indexed by the PANAS-SF include moods such as “interested” and “excited”. Engaging with loot boxes–mechanisms designed to promote interest and excitement–may therefore generally improve player mood. However, three very important caveats are warranted. First, to a large extent this is irrelevant. That some people have gambling problems does not mean that gambling is not fun, and that loot boxes are fun does not discount that they are disproportionately purchased by moderate risk and problem gambling populations, or that they share some fundamental psychological mechanisms with traditional forms of gambling. Second, and critically, the associations are quite small (*r* = .15), suggesting that the effect on mood is quite mild and may be of limited practical significance. Third, our hypotheses were directional and powered for one-tailed significance tests, so the fact that this relationship goes in the opposite direction to that predicted should be interpreted with considerable caution.

That non-randomized rewards were similarly associated with gambling symptoms is puzzling. The results are in direct contrast to Zendle & Cairns’ [7] findings that spending on non-randomized items was not associated with problem gambling symptomology. There are several potential ways of interpreting the finding. First, problem gambling symptoms may be related to a more general impulse control condition which results in players spending more on all forms of purchasing in video games. In this scenario, concerns about monetisation mechanisms in games may be unfounded, with control difficulties reflecting individual difference variables rather than game or reward system design elements. Second, the results may represent an intensification of the predatory monetization structures in games described by King and Delfabbro [26], and indicate that as randomized rewards and other monetization structures within games become increasingly entwined, players with higher problematic gambling symptomology find it harder to control purchasing for all reward types. Third, the effects may be a false positive result. Our data cannot discriminate between these options. Thus, we recommend further replications to specifically explore this issue.

We are incrementally constructing a picture of what the ‘at-risk’ user profile for high loot box engagement looks like. One novel finding of the present study is the fact that problem gambling symptoms and excessive gaming appear to combine in a meaningful fashion to produce a small [24, 27] association with loot box spending. This additive effect was slightly smaller than the effect of PGSI symptomology, and suggests that excessive gameplay may exacerbate risk for players with problem gambling symptoms. Thus, problem gambling symptoms are a risk factor for increased loot box spending, and this is especially true for those who engage in excessive gameplay. Although excessive gaming may help understand problematic loot box engagement, the present data emphasize the need to consider such behavior with a gambling framework [1, 11]. For a counterpoint see [16].

The present results also replicate Brooks and Clark’s general findings [5] that particular risky engagement with loot boxes are associated with higher spending on the mechanism. The Risky Loot Box Index was more strongly associated with spending on loot boxes than most other variables, suggesting that certain high-risk cognitions might be particularly important in understanding what drives excessive engagement with loot boxes. For instance, the specific risky cognitions assessed by the index include feeling compelled to open more boxes after the first, buying more loot boxes after not receiving valuable items, playing games longer than intended to specifically earn loot boxes, etc. Much as specific high risk gambling cognitions appear to contribute to higher spending on gambling [28, 29], specific risky cognitions appear to be associated with greater spending on loot boxes.

### Size of the effects

The observed effects were generally clear cut. The relationships between loot box spending behavior, problem gambling symptomology and risky loot box engagement were all of a size generally interpreted as practically significant, and similar when the effects of age and gender were statistically controlled. This replicates previous findings. Our work shows that the effects appear to be relatively similar in Australia, Aotearoa New Zealand, and the US, though further work with truly representative samples is required to confirm this.

While the relationship between loot box spending and problem gambling symptoms was non-trivial, smaller relationships were observed between loot box spending and negative mood and psychological distress [24, 27, 30]. Thus, although the associations between greater loot box spending and psychological distress and negative mood is undesirable, it is important to understand that the effects were relatively mild. Although people tended to feel a little worse when spending more on loot boxes, this is unlikely to produce practically meaningfully changes in users’ mood or distress. Moreover, it is important to note that, being a cross-sectional study, we are unable to determine causality. It may be that reverse causality is at play here; users who suffer increased psychological distress may purchase loot boxes in an attempt to alleviate distress. Further research is required to understand whether engagement with loot boxes is driving the small changes in psychological distress. We caution readers not to over interpret this result due to its small size and our present inability to determine causation.

Problem gamblers spent, on average, around $13 USD more per month on loot boxes than their peers who did not have such symptoms. This effect grew to approximately $21 USD per month when outliers were included in the analyses. This suggests that for the majority of users with gambling problems, the financial impact of engaging with loot boxes is relatively mild. However, the high standard deviations in the problem gambler group suggests that for a small number of users, spending increased much more than the $13 USD average (upper limit = $500 USD per month). Thus, it appears that some problem gamblers show a large increase in spending (relative to non-problem gamblers). As we discuss below, this may suggest that interventions designed to safeguard these high-spending users might be more appropriate than overarching policy initiatives. Similarly, it is important to put into context the actual amount of spending generated among problematic gambling users as, without context, it is likely that policy makers and the general public will assume the dollar amounts are far in excess of what they actually are. When considering policy interventions, the relatively small dollar amounts involved must also be weighed against any reasonably foreseen (restrictions of individual freedoms) and unforeseen consequences of government-led policy interventions. Additionally, it is important to reiterate that due to the failure of this study to collect a representative sample, further examination of the true average expenditure of the population of these countries on loot boxes is required.

### Cultural differences

Our exploratory analyses of the predictors of loot box spending across the three countries returned some interesting, albeit preliminary, results. Though we should interpret these exploratory results cautiously, the differences in effect size evident for the three countries may speak to cultural differences in gaming and gambling. The Aotearoa New Zealand sample showed the smallest relationship between problem gambling symptoms and loot box spending, suggesting that problem gambling symptoms are less closely aligned with loot box spending than in other countries. Aotearoa New Zealanders also showed the largest association between loot box spending and psychological distress, suggesting that there may be stronger psychological consequences to excessive loot box spending compared to the other countries. In contrast, Australia and the US had much stronger associations between loot box spending and problem gambling symptoms and weaker relationships between loot box spending and psychological distress. No country exceeded traditional cut-offs for a clinically meaningful effect, although the ANZ sample approached this effect size, warranting future investigations [24]. Further research should investigate whether national/cultural differences exist in the predictors and psychological consequence of loot box spending to aid in understanding whether an internationally unified or nation-specific approach to loot box policy is most appropriate.

### Policy implications

These results add to a growing body of evidence demonstrating that users with greater problem gambling symptomology spend more on loot boxes than those without such symptoms. This will encourage further debate around the most appropriate methods to limit such problems. In terms of the raw effect size, problem gamblers spent, on average around $13 USD more a month on loot boxes than their peers who did not have such symptoms (~$21 USD per month if outliers were included). Difference in spending behavior for moderate risk and problem gambler populations appears to be driven by a small number of high/extreme-spending users. Thus, harm minimization techniques designed to limit the financial harm experienced by these users may be one appropriate response to the issue. We therefore renew our call for the implementation of harm minimization interventions such as limit setting to reduce the likelihood of high-risk users experiencing financial harms from engagement with these systems [11]. As we have previously suggested, these techniques may be hard caps or self-set limits. For a variety of reasons, we believe it would be preferable that these take the form of industry-led initiatives. We note that some games companies have taken these concerns seriously, and are taking action to minimize the potential harm caused by these mechanisms. However, others have shown significant resistance to altering their monetization practices. We call on all games companies to seriously consider implementing harm minimization techniques such as limit setting to attenuate the potential financial harm that high-risk users may experience from loot box mechanisms.

### Limitations/future directions

The present manuscript reports a cross-sectional correlational study. Though it improves on earlier studies in a number of ways, it still cannot speak to issues of causation. Thus, we presently do not know the directionality of the relationships being investigated. Engagement with loot box systems may contribute to problem gambling symptoms or, more likely, problem gambling symptomology may be driving loot box spending. Better understanding the causal nature of the effects is a priority area for future research in this domain. If problem gambling symptoms are driving loot box spending, this would imply that there is little need to limit exposure to these systems for underage users and that limit setting policies might be an effective pathway to minimize harm for at-risk users. In contrast, if loot box spending were found to be causing the development of problem gambling symptoms, this would imply that exposure to such systems, especially for underage users, would be potentially hazardous.

Another limitation of the present study is that it investigates the relationships only in Western countries. There is significant discussion about the appropriateness of loot boxes in non-Western countries. For example, China and Japan have recently required odds disclosure for loot boxes to ensure that users know the chances of winning any particular item, and China are reportedly considering limiting the total number of loot boxes purchasable each day [2]. Further work is needed to understand whether the relationships between problem gambling symptomology, excessive game use, and loot box purchasing are consistent across a variety of countries and jurisdictions to adequately inform policymakers in each country of appropriate policy responses moving forward.

## Conclusion

People with greater problem gambling symptomology spend more on loot boxes than those without such symptoms, and those who engage in excessive gameplay and have problem gambling symptoms are at an even greater risk for high expenditure on loot box systems. These effects are relatively stable across a large sample in multiple Western countries. Our effects further highlight the psychological similarity between loot boxes and traditional modes of gambling, and suggest that for a small subset of high-risk users loot boxes may cause financial harm. We call on games companies to implement harm minimization techniques to limit the potential financial harm that high-risk users may experience from loot box mechanisms in video games.
